# A novel intra-operative technique to achieve accurate leg length and femoral offset during total hip replacement

**DOI:** 10.1308/003588412X13171221591259l

**Published:** 2012-05

**Authors:** S Alazzawi, SL Douglas, FS Haddad

**Affiliations:** University College London Hospitals NHS Foundation TrustUK

## BACKGROUND

Maintenance of leg length and femoral offset during a total hip replacement are known contributors to a satisfactory outcome.^1^ Various pre and intra-operative methods to achieve this are described in the literature.^2^ Here we add a simple, quick and reliable intra-operative technique.

## TECHNIQUE

**Figure 1:**
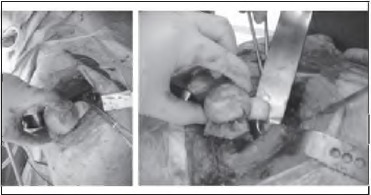
The surgeon aligns the trial head and neck implants with the osteotomised femoral head to match the anatomical femoral offset and leg length.

We recommend pre-operative templating as routine practice prior to hip replacement surgery. During the procedure, the femoral neck cut is performed based on clinical judgement and the pre-operative template measurements. The femur is prepared as usual and the final rasp is used as a trial femoral implant. At this stage, a series of head and neck implants of various sizes and offsets are usually tested to establish the required size of the final prosthesis. Our technique involves comparing the trial head and neck implants with the osteot- omised femoral head using visual assessment. This can be achieved by placing the osteotomised femoral head adjacent to the neck cut (Fig 1). It then allows the surgeon either to match the centres of the prosthetic and original heads or increase/decrease the offset as required. The same step can be repeated after inserting the final femoral stem to confirm the correct size of prosthetic head to be used.

**Figure 2:**
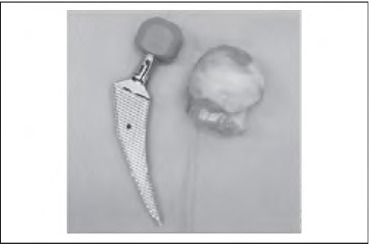
Checking compatibility between the osteotomised femoral head and neck and the implant components

## DISCUSSION

While most of the previously described intra-operative techniques require insertion of pins, the use of intra-operative x-ray or additional kits, our technique is a safe and non-invasive method that requires no additional equipment, cost or time (Fig 2).
